# Quantitative analysis of morphological and functional alterations of the meibomian glands in eyes with marginal entropion

**DOI:** 10.1371/journal.pone.0267118

**Published:** 2022-04-14

**Authors:** Min Kyu Yang, Ho-Seok Sa, Namju Kim, Hyun Sun Jeon, Joon Young Hyon, Hokyung Choung, Sang In Khwarg

**Affiliations:** 1 Department of Ophthalmology, Asan Medical Center, University of Ulsan College of Medicine, Seoul, Korea; 2 Department of Ophthalmology, Seoul National University College of Medicine, Seoul National University Bundang Hospital, Seongnam, Korea; 3 Department of Ophthalmology, Seoul Metropolitan Government-Seoul National University Boramae Medical Center, Seoul, Korea; 4 Department of Ophthalmology, Seoul National University College of Medicine, Seoul National University Hospital, Seoul, Korea; University of Toronto, CANADA

## Abstract

**Purpose:**

To quantitatively analyze morphological and functional alterations of the meibomian glands in eyes with marginal entropion and their changes after surgery.

**Methods:**

Sixty eyes of 52 patients with marginal entropion and underwent meibography and interferometer were included. One-hundred and seventeen age- and sex-matched eyes with minimal to mild meibomian gland dysfunction (MGD) were recruited as control eyes. Meibomian gland loss (MGL) and lipid layer thickness (LLT) were compared between eyes with marginal entropion and control eyes. Subgroup analysis was performed according to the extent of entropion. MGL and average LLT at 1 and 5 months after surgery were compared with those of 20 eyes with marginal entropion followed without surgery.

**Results:**

In eyes with marginal entropion, MGL was higher (27.7% vs. 12.8%, P = 0.014), and average LLT was thinner (64 nm vs. 86 nm, P = 0.005) than those in control eyes. MGL was higher in eyes with more extensive entropion (> 2/3 eyelid width) than in eyes with less extensive entropion (≤ 1/3 eyelid width) (40.5% vs. 13.2%, P = 0.001). Average LLT increased after surgery (97 nm at 1 month, P = 0.003; 75 nm at 5 months, P = 0.319), and thicker than that of eyes followed without surgery (97 nm vs. 66 nm, P = 0.046). MGLs after surgery remained unchanged from the preoperative MGL (all P > 0.7).

**Conclusion:**

Marginal entropion is associated with morphological and functional alterations of the meibomian glands. Functional improvement after entropion repair suggests that marginal entropion could cause or exacerbate MGD. Further studies are required to establish the role of entropion repair in managing MGD.

## Introduction

Marginal entropion is a mild form of cicatricial entropion accompanied by subtle inward rotation of the eyelid margin [1]. Rounded posterior eyelid margin and anterior migration of the mucocutaneous junction without obvious cicatricial changes of the tarsal conjunctiva indicate a diagnosis of marginal entropion [2,3]. Surgical correction using eyelid margin splitting and anterior lamellar repositioning has excellent clinical outcomes for marginal entropion [1].

Structural and functional alterations of the meibomian glands (MGs) have been reported in studies of entropion [4–7]. Keratinized lid margin frequently occurs in cicatricial entropion, resulting in dislocation of the MG orifice [7]. The MGs in the cicatricial entropion exhibit partial atrophy of the gland acini and chronic inflammatory cell infiltration [4]. The number of functioning MGs was lower in eyes with involutional lower eyelid malposition than in normal eyelids [6]. Anteriorization of the Marx line was strongly correlated to meiboscore and MG secretion score [5]. However, quantitative and objective evaluation, and the effect of entropion repair on the MGs, have not been assessed in prior studies.

The LipiView^®^ ocular surface interferometer (TearScience Inc., Morrisville, NC, USA) automatically measures incomplete blinking (IB) and lipid layer thickness (LLT) [8]. It also provides noncontact meibography enhanced by dynamic illumination, allowing a rapid and patient-friendly assessment of the MG structure and MG dropout area [8,9]. In the present study, we evaluated morphological and functional alterations of the MGs in eyes with marginal entropion and their changes after entropion repair, using noncontact meibography and interferometer.

## Methods

We retrospectively reviewed the electronic medical records of patients with marginal entropion and underwent meibography and interferometer at Seoul National University Bundang Hospital from November 2016 to December 2021. The diagnosis of marginal entropion was established in the case of constantly rolled posterior eyelid margin and anterior migration of the mucocutaneous junction beyond the MG orifices with no or minimal cicatricial change of the tarsal conjunctiva, as evaluated by slit-lamp biomicroscopy. Exclusion criteria were conditions that affect LLT or MG dropout, such as structural changes of the ocular surface (for example, pterygium), active inflammation of the eyelid and ocular surface (for example, chalazion or conjunctivitis) during the entire follow-up period, thyroid eye disease, use of anti-glaucoma eye drops or contact lens more than 1 year, or a history of ocular surgery performed within 2 years [10–13]. Trichiasis and obvious cicatricial change of the tarsal conjunctiva were also included in the exclusion criteria.

Age- and sex-matched patients having minimal to mild meibomian gland dysfunction (MGD), i.e. mildly altered meibomian secretions and MG expressibility, were recruited as a control cohort. Two ocular surface disease specialists (HSJ, JYH) diagnosed based on following criteria [14]: meibum score < 8 in 8 glands of the central third of the lower eyelid on a scale of 0 to 3 for each gland, and 3 to 4 glands expressible in 5 glands in the lower or upper eyelid. All control eyes presented no eyelid malposition and had meibographic and interferometric results. The study was conducted according to the tenets of the Declaration of Helsinki. The study protocol was approved by the institutional review board of Seoul National University Bundang Hospital (IRB No. B-2101-660-106).

At the initial visit, meibography and interferometer were performed, and medical photographs of the eyelid margins were obtained in the examination room with dim light (20–30 lx), room temperature (22°C ± 2°C) and humidity (30% ± 5%). Patients were firmly positioned on the forehead and chin rest of the device. In the lipid imaging mode, patients were instructed to maintain fixation on the internal target and blink naturally for 20 s [8]. After focusing on the tear film surface slightly below the inferior pupillary margin, LLT was quantified based on the dominant color of the interference patterns in the specular reflection zone for the period of observation [15]. LLT was measured as the maximum, minimum, and average values over 20 s in the nanometer. IB and total blinking were also measured during the same period. IB ratio was defined as the value of IB divided by total blinking.

Immediately after LLT measurement, noncontact meibography using infrared light was performed in the Gland Imaging Mode. An experienced examiner carefully everted the upper and lower eyelids with a handheld lid everter to expose the entire tarsal area. Meibography images were exported in JPEG format and evaluated with ImageJ software (Version 1.52h, US National Institutes of Health, Bethesda, MD, USA). MG dropout area was calculated using the polygon selection tool. Meibomian gland loss (MGL) was defined as the proportion of the MG dropout area in the central 3/4 of the upper and lower tarsal areas [8,16]. The mean values of three repeated measurements made by an ophthalmologist (MKY) were used for further analysis. In medical photographs, the extent of entropion was measured as the width of the mucocutaneous junction beyond the MG orifices.

We routinely recommended eyelid margin splitting and anterior lamellar repositioning as a surgical correction, but some patients refused and were followed without intervention. Surgical correction was performed by a single oculoplastic surgeon (NK), and its detailed techniques were described in our previous study [1]. Briefly, a 1 or 2 mm-deep incision was made along the gray line in accordance with the horizontal extent of the lower eyelid marginal entropion (eyelid margin splitting). After subciliary skin incision and dissection between the orbicularis and tarsus, several double-armed 6–0 Vicryl sutures (Ethicon Inc., Somerville, NJ, USA) were placed in the middle of the tarsus. Both arms of the sutures were passed through the anterior lamellar and subsequently were securely tied just below the eyelashes (anterior lamellar repositioning). Topical antibiotic ointment was applied to the wound and exposed sutures for 1 week. Postoperative follow-up with interferometer measurement was routinely performed at 1 month after surgery, and 5 months after surgery in some cases. The ocular surface, eversion of the eyelid margin, and direction of cilia were examined by slit-lamp biomicroscopy at each follow-up. The anterior lamellar repositioning suture knots were removed 1 month postoperatively.

Demographics, results of meibography and interferometer were compared between eyes with marginal entropion and control eyes. The prevalence of average LLT ≤ 60 nm was also compared, as this cut-off value best correlated with MGD and severe dry eye symptoms [15,17]. Meibographic and interferometric results were compared between both eyes of patients that had unilateral marginal entropion. Eyes with marginal entropion were divided into three groups according to the extent of entropion (≤ 1/3 eyelid width, 1/3~2/3 eyelid width, > 2/3 eyelid width), to compare MGL and average LLT using the Kruskal–Wallis test and Dunn’s post hoc test with Bonferroni correction. Postoperative MGL and average LLT at 1 and 5 months were also compared to MGL and average LLT measured twice with an interval of 3~6 months in eyes with uncorrected marginal entropion. Statistical analyses were performed using the Wilcoxon test or Mann–Whitney U test for continuous variables and chi-square test for categorical variables in MedCalc software (version 9.6.4.0, MedCalc Software, Mariakerke, Belgium). A P value < 0.05 was considered statistically significant.

## Results

This study included 19 males (20 eyes) and 33 females (40 eyes) with marginal entropion. Eight patients (13.3%) had bilateral involvement. The upper eyelid was involved in twelve eyes (20.0%), while the lower eyelid was involved in 48 eyes (80.0%). Seventeen eyes (28.3%) had an extent of ≤ 1/3 eyelid width, 24 eyes (40.0%) had an extent of 1/3~2/3 eyelid width, and 19 eyes (31.7%) had an extent of > 2/3 eyelid width. Minimal cicatricial change of the tarsal conjunctiva was observed in five patients (seven eyes). However, the possible etiology of cicatricial changes such as pemphigoid, atopic dermatitis, or Stevens–Johnson syndrome was not identified in the medical records.

Demographics and meibographic and interferometric results in eyes with marginal entropion and control eyes are shown in Table 1. The degree of corneal erosion was mild (less than ten erosions) in all patients. Proportions of eyes underwent cataract surgery before entropion surgery were similar (36.7% in eyes with marginal entropion vs 30.0% in control eyes, P = 0.398), and no patient had a history of previous eyelid surgery. MGL was significantly higher in eyes with marginal entropion than in control eyes (27.7% vs. 12.8%, P = 0.014, Mann–Whitney U test). IB ratio was similar in eyes with marginal entropion and control eyes (47.2% vs. 50.0%, P = 0.222). Maximum, minimum, and average LLT were significantly thinner in eyes with marginal entropion than in control eyes (average LLT: 64 nm vs. 86 nm, P = 0.005, Mann–Whitney U test). In addition, the prevalence of average LLT ≤ 60 nm was higher in eyes with marginal entropion than in control eyes (45.0% vs. 28.2%, P = 0.030, chi-square test). In a subgroup analysis of upper eyelid entropion, all meibographic and interferometric results were similar to those of lower eyelid entropion with statistical significance in comparing MGL and minimum LLT.

**Table 1:** Demographics, results of meibography and interferometer in eyes with marginal entropion and control eyes.

		Eyes with marginal entropion	Control eyes	P value^a^
Eyes (patients), No.		60 (52)	117 (60)	
Male, No. (%)		19 (31.7)	49 (41.9)	0.197
Age at surgery, years		70.6 (64.3–77.5)	67.7 (57.3–77.6)	0.394
Meibomian gland loss, %				
Total (upper and lower)		27.7 (13.7–43.6)	12.8 (8.0–21.5)	**< 0.001**
Upper eyelid entropion		37.8 (11.1–45.6)		**0.014**
Lower eyelid entropion		27.6 (13.7–43.4)		**< 0.001**
Lipid layer thickness (LLT), nm				
Maximum	Total (upper and lower)	85 (61–100)	100 (80–100)	**0.002**
	Upper eyelid entropion	85 (75–100)		0.074
	Lower eyelid entropion	85 (60–100)		**0.006**
Minimum	Total (upper and lower)	43 (30–62)	59 (42–83)	**< 0.001**
	Upper eyelid entropion	41 (34–56)		**0.041**
	Lower eyelid entropion	43 (27–62)		**< 0.001**
Average	Total (upper and lower)	64 (48–98)	86 (58–100)	**0.005**
	Upper eyelid entropion	69 (51–84)		0.117
	Lower eyelid entropion	62 (46–100)		**0.006**
Average LLT ≤ 60 nm, No. (%)		27 (45.0)	33 (28.2)	**0.030**

Data are presented as the median (interquartile range), and values in bold indicate statistical significance.

^a^Mann–Whitney U test for continuous variables and chi-square test for categorical variables.

**Fig 1:**
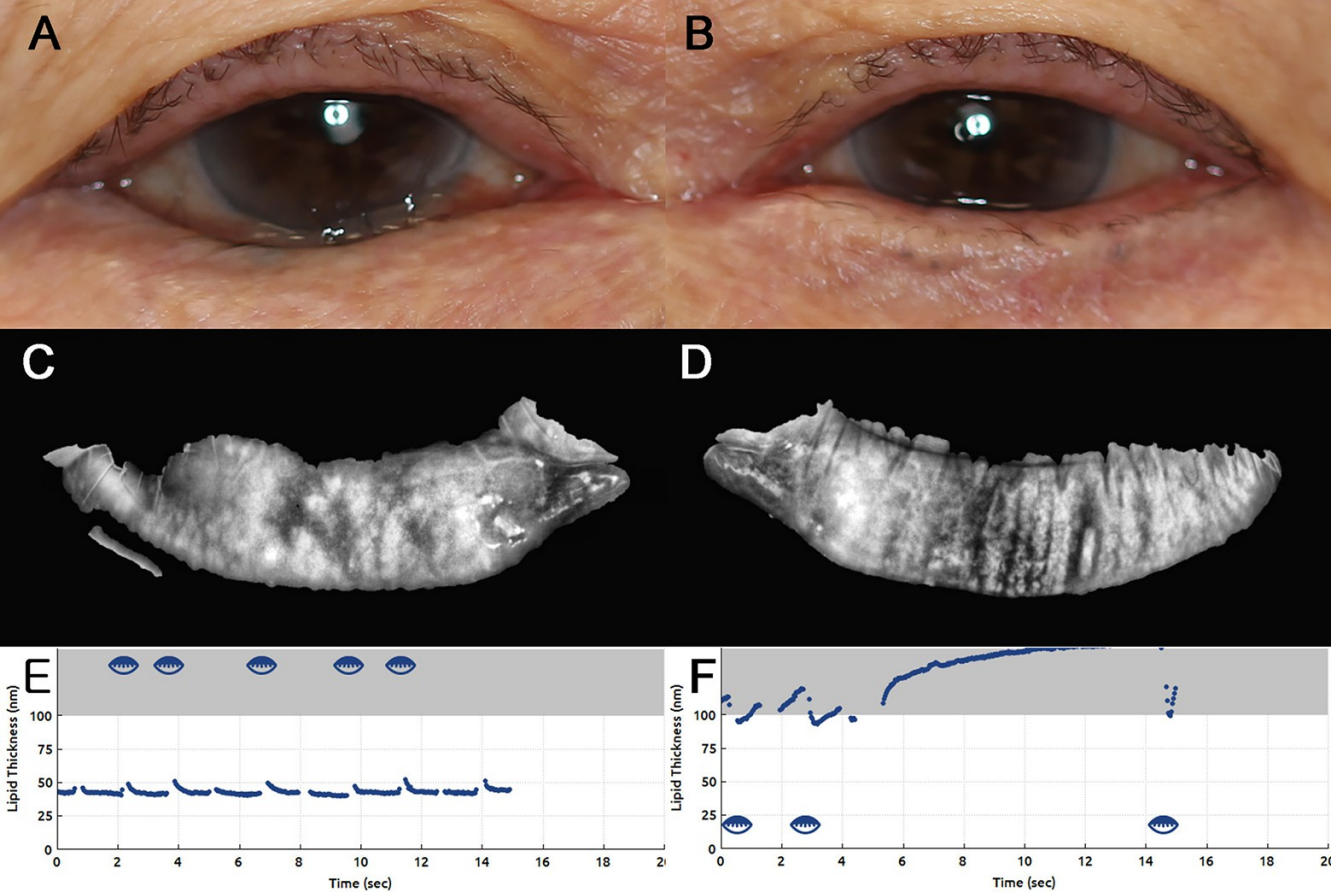
An 82-year-old woman with unilateral lower eyelid marginal entropion showed asymmetric meibomian gland profiles. (A, B) The right lower eyelid had marginal entropion over the entire length, while the left had no entropion. (C, D) Meibomian gland dilation and dropout were observed in the right lower eyelid (meibomian gland loss: 17.7% in the right eye vs. 7.0% in the left eye). (E, F) Average lipid layer thickness was thinner in the right eye (43 nm vs. > 100 nm).

In patients with unilateral entropion, MGL was higher and LLT was thinner in eyes with entropion than in contralateral normal eyes (Table 2), which was similar to the comparison between eyes with marginal entropion and control eyes. A representative case is shown in Fig 1, an 82-year-old woman with unilateral lower eyelid marginal entropion presenting with asymmetric MG profiles.

**Table 2:** Comparisons of meibographic and interferometric results between eyes with unilateral marginal entropion and contralateral normal eyes.

n = 44	Eye with entropion	Contralateral eye	P value^a^
Meibomian gland loss, %	28.0 (15.9–45.6)	17.1 (7.9–27.5)	**0.002**
Incomplete blinking ratio, %	33.3 (0.0–66.7)	20.0 (0.0–50.0)	0.390
Lipid layer thickness, nm			
Maximum	88 (60–100)	100 (92–100)	**0.007**
Minimum	39 (29–52)	58 (45–73.0)	**0.002**
Average	68 (48–84)	87 (64–100)	**0.005**

Data are presented as the median (interquartile range), and values in bold indicate statistical significance.

^a^Wilcoxon test.

**Fig 2:**
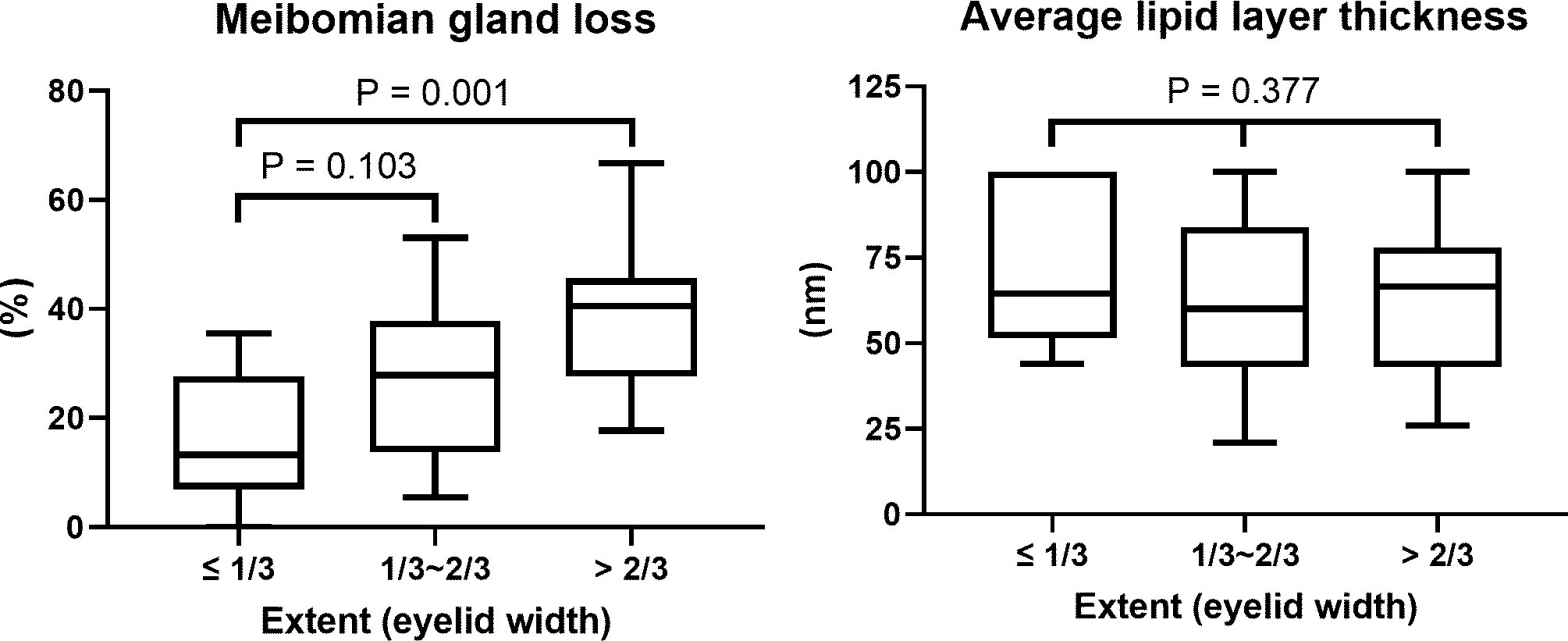
Box-and-whisker plots of meibomian gland loss and average lipid layer thickness according to entropion extent. Meibomian gland loss was significantly higher in eyes with entropion extent > 2/3 eyelid width than in eyes with entropion extent ≤ 1/3 eyelid width (median 40.5% vs. 13.2%, P = 0.001, Dunn’s post hoc test with Bonferroni correction).

In a comparison of MG profiles according to the extent of marginal entropion (≤ 1/3, 1/3~2/3, > 2/3 eyelid width), there was a significant difference in MGL among the three groups but not in average LLT (P = 0.002 and 0.377, respectively, Kruskal–Wallis test). MGL was significantly higher in eyes with more extensive entropion (> 2/3 eyelid width) than in eyes with less extensive entropion (≤ 1/3 eyelid width) (median 40.5% vs. 13.2%, P = 0.001, Dunn’s post hoc test with Bonferroni correction, Fig 2).

Successful surgical outcomes for entropion correction were achieved in all 40 eyes, defined as no residual cilia touching the ocular surface with complete resolution of corneal erosions. Slit-lamp biomicroscopy revealed outward rotation of the entire eyelid margin and MG orifice in all eyelids. All 34 patients were satisfied with the cosmetic results, and surgical complications were not observed in any cases. Twenty eyes of 18 patients were followed without surgery, and the median interval between interferometry was 3.6 (2.9–5.8) months. Demographics were similar in eyes underwent surgery and in eyes followed without surgery (all P > 0.4).

**Fig 3:**
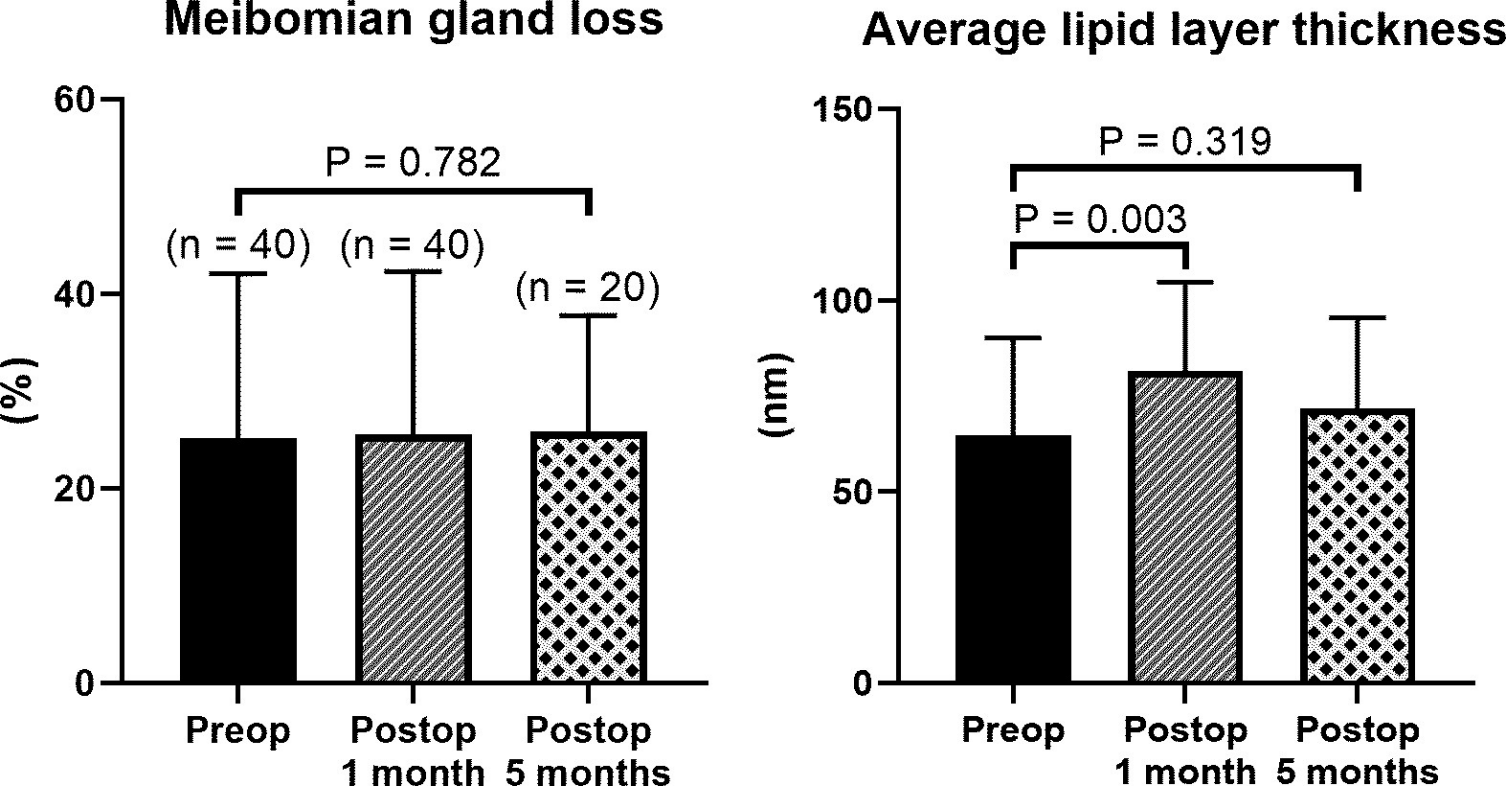
Changes of meibomian gland loss and average lipid layer thickness after surgery for marginal entropion. Meibomian gland loss at 1 and 5 months after surgery remained unchanged from preoperative meibomian gland loss (24.2% vs. 23.0% at 1 month and 24.2% at 5 months after surgery, respectively, all P > 0.05). Average lipid layer thickness increased after surgery (63 nm vs. 97 nm at 1 month and 75 nm at 5 months after surgery, respectively) with statistical significance at 1 month after surgery (P = 0.003).

MGL and average LLT in eyes followed without surgery remained unchanged between the follow-ups (all P > 0.99). In contrast, average LLT increased in eyes underwent surgical correction. At 1 month after surgery, average LLT significantly increased (63 nm vs. 97 nm, P = 0.003, Wilcoxon test; Fig 3). It was thicker than average LLT in eyes followed without surgery (97 nm vs. 66 nm, P = 0.046). At 5 months after surgery, LLT and MGL were obtained for 20 eyes. Average LLT decreased compared with LLT at 1 month postoperatively, and there was no significant difference from the preoperative LLT (63 nm vs. 75 nm, P = 0.319, Mann–Whitney U test). The prevalence rate of average LLT > 90 nm at 5 months after surgery was higher than before surgery with marginal significance (50.0% vs. 25.0%, P = 0.081). MGLs at 1 and 5 months after surgery remained unchanged from the preoperative MGL (24.2% vs. 23.0% at 1 month and 24.2%, at 5 months after surgery, respectively, all P > 0.7).

In patients with unilateral marginal entropion, similar trends of postoperative average LLT changes were observed (70 nm vs. 100 nm at 1 month and 98 nm at 5 months after surgery, respectively) with statistical significance at 1 month after surgery (P = 0.012). There were no significant differences in average LLT between eyes with marginal entropion and contralateral normal eyes throughout postoperative follow-ups (100 nm vs. 100 nm at 1 month, P = 0.831; 86 nm vs. 98 nm at 5 months, P > 0.99, respectively).

## Discussion

The present study quantitatively analyzed morphological and functional alterations of the MGs in eyes with marginal entropion by assessing MGL and LLT measured by noncontact meibography and interferometer. MGL was higher and LLT was thinner in eyes with marginal entropion than in those of control eyes. More extensive entropion was associated with higher MGL. After entropion repair, LLT increased but MGL remained unchanged throughout the 5-month follow-up.

Prior studies have described MG changes in eyes with entropion [4–7], but have not provided objective evaluations. Our study is the first to objectively evaluate the MG profiles in eyes with entropion. Higher MGL and thinner LLT in eyes with marginal entropion suggest that marginal entropion is associated with obstructive MGD [8,18]. Similarly, cicatricial entropion has been widely accepted as a major etiologic factor for obstructive MGD [19]. However, evaluating the MG profiles in cicatrized conjunctiva has limitations to prove association between entropion and MGD. Conjunctival scarring hinders eversion of the eyelid and clear visualization of the MGs, compromising accurate measurement of MGL. Conjunctival scarring can also directly affect MG structure itself. The decreased LLT may be due to eyelid malposition, but may also be due to altered MG structure. Hence, we investigated MG profiles in eyes with marginal entropion, which is characterized by abnormal location of the MG orifices at the eyelid margin without obvious cicatricial changes of the tarsal conjunctiva.

Because aging and female sex are associated with increased MGL and decreased LLT [18,20,21], we constituted a control cohort that was age- and sex-matched. Controls were patients with minimal to mild MGD without entropion who underwent meibography and interferometer, as interferometric data from normal elderly persons were rare in our institute. Clinical findings of controls were compatible with obstructive MGD. Therefore, it can be assumed that the LLT of normal subjects is not thinner than that of controls [18], and is much thicker than that of eyes with marginal entropion. Additionally, we compared eyes with entropion to normal contralateral eyes in unilateral entropion patients. These two comparisons equally showed higher MGL and thinner LLT in eyes with marginal entropion. In the subgroup analysis of upper or lower eyelid entropion, both subgroups showed higher MGL and thinner LLT than in controls, but the statistical significance was insufficient in eyes with upper eyelid entropion. It is probably due to the small number of eyes with upper eyelid entropion, because the values of MGL and LLT were similar in both subgroups.

In contrast to MGL, which was quantitatively correlated with the extent of marginal entropion, LLT was not correlated with the extent of marginal entropion. This could be explained by compensatory overproduction of tear film lipids in the remaining MGs. A similar homeostatic system with overproduction of tear fluid to compensate for the loss of MGs has been proposed by Arita et al. [22].

Another major finding of the present study is the increase of LLT after surgical correction of marginal entropion. Siah et al. [23] postulated that marginal entropion is a result of MGD, as the chronic subclinical inflammatory processes of MGD result in tarsal contraction and MG inversion. Our finding supposes that marginal entropion could also cause or exacerbate MGD. Persistent contact between MG orifices and the ocular surface could aggravate corneal and conjunctival epithelial cell shedding and obstruction of the MG orifice. Distorted MG ducts could inhibit the secretion of meibum [23]. Damming back of MG secretions causes inflammation and cystic dilatation of the duct, and ultimately atrophy of the acini. After surgical correction, repositioning of MG ducts and orifices would facilitate release of pooled secretions, increasing LLT.

Increased average LLT at 1 month after surgery tended to decrease again at 5 months. This could potentially be explained by diminished overproduction of tear film lipids or that the pooled secretion was depleted over time after the resolution of MG obstruction. Nontheless, the prevalence rates of average LLT > 90 nm were still as high as two times, and the small numbers of patients might be contributed to the marginal significance. Eyelid margin splitting procedure is considered to not to compromise tarsoconjunctiva and meibomian glands [24]. We also observed that the carefully performed eyelid margin splitting procedure did not appear to have a harmful effect on the MGs, as there was no change in MGL after surgery for up to 5 months.

Decreased LLT is correlated with signs and symptoms of dry eye syndrome and MGD [8,15,17,25]. LLT increase after meibomian therapy is associated with improvement in ocular comfort [26]. Therefore, surgical correction of marginal entropion could have additional benefits in improving dry eye syndrome and MGD. Similarly, Siah et al. [23] reported improved signs and symptoms of MGD after corrective surgery for meibomian gland inversion. The effect of entropion repair for dry eye syndrome and MGD is thought to be sustained throughout the 5-month follow-up. Although there was no statistical significance, average LLT at 5 months after surgery was still higher than the preoperative value. The prevalence rates of average LLT ≤ 60 nm at 1 and 5 months after surgery were similar to that of control eyes.

In eyes with entropion, epiphora can occur due to ocular irritation and reflex lacrimal hypersecretion [27]. Elevated tear meniscus can affect LLT: LLT increases after surgery for nasolacrimal duct obstruction [28,29]. Therefore, there is a possibility of the LLT increase after entropion repair in the present study is a result of epiphora resolution, instead of improved MGD. However, the cilia touching width was much less extensive than the width of mucocutaneous junction beyond the MG orifices, and the degree of corneal erosion was mild in our subjects. LLT was measured under dim light to minimize reflex tearing. In these conditions, the effect of reflex epiphora on LLT could be small.

There are several limitations to our study that should be acknowledged in order to avoid its overinterpretation. First, the retrospective study had a small cohort of Asian patients, and the follow-up period was only 5 months. Second, a symptom questionnaire (for example, ocular surface disease index), which is important in MGD diagnosis and treatment response, was not obtained. However, even if conducted, the results of this questionnaire would not be able to distinguish between ocular discomfort due to ciliary touching or MGD. Instead, we showed a higher rate of average LLT ≤ 60 nm in eyes with marginal entropion, which is known to be associated with severe dry eye symptoms [15]. To fully confirm the relationship between marginal entropion and MGD, larger long-term prospective worldwide studies including multiple ethnicities that also evaluate meibum characteristics and dry eye status are required.

In conclusion, marginal entropion is associated with morphological and functional alteration of the MGs. Functional improvement after entropion repair suggests that marginal entropion could cause or exacerbate MGD. Further studies are required to establish the role of entropion repair in managing MGD.
